# Maternal blood metal concentrations are associated with C-reactive protein and cell adhesion molecules among pregnant women in Puerto Rico

**DOI:** 10.1097/EE9.0000000000000214

**Published:** 2022-07-05

**Authors:** Christine Kim, Amber L. Cathey, Deborah J. Watkins, Bhramar Mukherjee, Zaira Y. Rosario-Pabón, Carmen M. Vélez-Vega, Akram N. Alshawabkeh, José F. Cordero, John D. Meeker

**Affiliations:** a Department of Environmental Health Sciences, University of Michigan School of Public Health, Ann Arbor, Michigan; b Department of Biostatistics, University of Michigan School of Public Health, Ann Arbor, Michigan; c University of Puerto Rico Graduate School of Public Health, UPR Medical Sciences Campus, San Juan, Puerto Rico; d College of Engineering, Northeastern University, Boston, Massachusetts; e Department of Epidemiology and Biostatistics, University of Georgia, Athens, Georgia.

**Keywords:** metals, inflammation, VCAM, ICAM, CRP, pregnancy, Puerto Rico

## Abstract

Studies have revealed a link between aberrant levels of maternal C-reactive protein (CRP) and cell adhesion molecules (CAMs) with adverse birth outcomes. Some epidemiologic studies have indicated that long-term metal exposures can modulate the levels of CRP and CAMs, but the associations between prenatal metal exposures and the levels of CRP and CAMs have yet to be studied more extensively. In this study, we assessed associations between maternal blood metal levels and CRP/CAMs among 617 pregnant women in the Puerto Rico PROTECT birth cohort.

**Methods:**

Blood samples were collected from participants at 16–20 (visit 1) and 24–28 (visit 3) weeks gestation. We measured concentrations of 11 metals using inductively coupled plasma mass spectrometry (ICP-MS). From the blood samples, CRP and CAMs intercellular adhesion molecule (ICAM) and vascular cell adhesion molecule (VCAM) were also quantified using a customized Luminex assay. Linear-mixed effects models (LMEs) were used to regress CRP and CAMs on metals and included random intercepts for study participants to account for correlated repeated outcome measures. Fetal sex and visit effects were estimated using interaction terms between metal exposure variables and fetal sex, as well as visit indicators, respectively.

**Results:**

We observed significant positive associations between nickel and CRP (Δ: 7.04, 95% CI = 0.75, 13.73) and between lead and VCAM (Δ: 4.57, 95% CI = 1.36, 7.89). The positive associations were mainly driven by mothers carrying male fetuses. We also observed various visit-specific associations. The significant associations between metals and CRP were predominantly driven by visit 3; however, the significant associations between metals and VCAM were mainly driven by visit 1.

**Conclusion:**

Certain maternal blood metal levels were significantly associated with CRP and CAMs and most of these associations were differentially driven by fetal sex, as well as by timing in pregnancy. Future studies should further explore metal-CRP/CAMs associations for a better understanding of the underlying mechanism of metal-induced adverse birth outcomes.

**What this study adds**

Several previous studies have observed an increased risk of adverse birth outcomes with elevated levels of CRP and CAMs. However, there is a lack of research on elucidating associations between prenatal metal exposures with CRP and CAMs. This study highlights significant metal-CRP/CAMs associations that are fetal sex- and visit-specific and may inform research on new avenues for understanding metal-induced adverse birth outcomes.

## Introduction

Many toxicological studies suggest that heavy metal exposure leads to oxidative stress and inflammatory response and modulates the expression of inflammatory markers.^1-4^ Importantly, some human studies have demonstrated a link between heavy metal exposure and inflammatory markers, such as C-reactive protein (CRP), intercellular adhesion molecule (ICAM), and vascular cell adhesion molecule (VCAM).^5,6^ These inflammatory markers play important roles in inflammatory processes and recruiting leukocytes during an inflammatory response.^7,8^ Proinflammatory cytokines, such as IL-1β and TNFα, are known to regulate expression levels of CRP, ICAM, and VCAM,^9-11^ and toxicologic studies have consistently demonstrated that heavy metal exposure increases these proinflammatory cytokine levels,^12-14^ further emphasizing the association between heavy metal exposure and inflammatory markers.

Pregnancy is a dynamic process during which multiple inflammatory events occur, especially during implantation, placentation, and preparation for delivery.^15,16^ Therefore, it is common to observe dynamic changes in the inflammatory profile during normal pregnancy.^15,17^ Even though the exact role of CRP and CAMs in pregnancy is not yet defined, studies have observed an increased risk of adverse birth outcomes, such as preeclampsia and preterm birth, with elevated levels of CRP and CAMs.^18-29^ Notably, Grgic et al^21^ observed that CRP levels greater than 4 mg/L are linked to the development of premature birth. Similarly, there were significant increases in VCAM and ICAM levels in preeclampsia, as compared to normal pregnancy in South Korea (n = 178).^29^ Together, these studies highlight the importance of a fine balance of both CRP and CAMs for healthy pregnancies. Only limited studies have investigated the role of inflammatory profiles in pregnancy, which highlights the complexity of fully understanding adverse birth outcomes that arise from an imbalance of inflammatory markers.

The associations between heavy metal exposures and adverse birth outcomes, such as preterm birth and preeclampsia, are well established.^30-33^ For example, epidemiologic studies have demonstrated positive associations between Pb and Cd exposures with the incidence of preterm birth.^32^ However, the mechanisms by which metal exposures impact birth outcomes are not clearly understood. Currently, available data suggest possible mechanisms, one of which includes inflammation.^32,34^ Because there are dynamic changes in the inflammatory profile during normal pregnancy, there is a growing interest in dissecting the effects of metal exposure on the inflammatory profile that contribute to adverse birth outcomes. Further, a paucity of research on the association between metal exposures with CRP and CAMs levels in humans, particularly during pregnancy, necessitates more investigations. The overarching goal of this study was to investigate associations between prenatal metal exposures with CRP and CAMs expression levels among pregnant women in the ongoing Puerto Rico PROTECT cohort. We believe this study will improve the current understanding of the impact of prenatal metal exposures on maternal inflammatory markers.

## Methods

This study sample is a subset of the Puerto Rico PROTECT cohort. The PROTECT cohort began recruitment in 2010 through funding from the National Institute of Environmental Health Sciences Superfund Research Program. PROTECT has built detailed and extensive data sets on environmental conditions as well as prenatal conditions (close to 3,000 data points per participant) of 1,800 pregnant women in Puerto Rico’s North Coast. Because Puerto Rico has a large number of Superfund (18 Superfund sites) and other hazardous waste sites in Northern karst regions, the study participants may be exposed to elevated levels of a range of Superfund-relevant chemicals, including metals. Rates of adverse pregnancy outcomes, including preterm birth, in Puerto Rico are among the highest of all US states and territories. Study participants were recruited in the first or early second trimester of pregnancy (median 14 weeks gestation) at seven prenatal clinics and hospitals throughout Northern Puerto Rico during 2011–2019. Women visiting the clinic for their first prenatal visit who may meet eligibility criteria were told briefly about the study by their nurse or physician at participating hospitals and were given a brochure that describes the study in general terms. Study nurses then approached eligible patients during their next prenatal visit (typically before 16 weeks). Informed, signed consent was obtained from each eligible woman interested in participating. Participating women were then followed up at four additional times during data and samples are collected: visit 1 (between 16–20 weeks gestation), visit 2 (20–24 weeks), visit 3 (24–28 weeks), and at delivery. V1 and V3 took place at the clinics, where brief questionnaires were given and blood and urine samples were collected. V2 took place at the participants’ home, where tap water, urine, and hair samples were collected. Inclusion criteria for recruitment included: participant age between 18 and 40 years; residence in the Northern Karst aquifer region; disuse of oral contraceptives 3 months before pregnancy; disuse of in vitro fertilization; and no indication in self report for major obstetrical complications, including pre-existing diabetes. Each participant completed a nurse-administered baseline questionnaire that collects information on variables such as demographic data, alcohol consumption, drug use, socioeconomic status indicators, work history, age at menarche, and information on medical risk factors for adverse pregnancy outcomes. In addition, the questionnaire acquired information on residential history and self-reported exposures to a number of environmental agents such as secondhand tobacco smoke, pesticides, and others. The recruitment rate as of 2021 was 97% and the loss to follow up is low in PROTECT. This study was approved by the research and ethics committees of the University of Puerto Rico, Northeastern University, and participating hospitals and clinics. All methods reported in this study were performed in accordance with relevant guidelines and regulations imposed by those institutions. All study participants provided full-informed consent before participation.

### Blood biomarker measurements

Whole blood samples were collected at two study visits (median 18 [visit 1] and 26 weeks [visit 3] gestation). Timing for the visits was based on a number of factors, including the ability to coincide with routine clinic visits to minimize costs and participant burden and to align with periods of rapid fetal growth. Samples were collected in metal-free tubes, divided into aliquots, frozen at –80°C, and shipped on dry ice to NSF International (Ann Arbor, MI, USA) for analysis. The whole blood samples were used to measure blood concentrations of metals, as well as CRP and CAMs.

### Blood metals

Blood concentrations of metals were measured using a Thermo Fisher’s (Waltham, MA, USA) iCAP RQ inductively coupled plasma mass spectrometry (ICP-MS) and CETAC ASX-520 autosampler, as described previously.^35^ Standards of known purity and identity were used during the preparation of the calibration, quality control, and internal standards. The ICP-MS was calibrated with a blank and a minimum of four standards for each element of interest. The calibration curve response versus concentration was evaluated for the goodness of fit. All validated analyte correlation coefficients (R) were ≥0.995. Eleven metals were included in our analysis: cadmium (Cd), cesium (Cs), cobalt (Co), copper (Cu), lead (Pb), manganese (Mn), mercury (Hg), molybdenum (Mo), nickel (Ni), tin (Sn), zinc (Zn). Of these 11 metals, Co, Cu, Mn, Mo, and Zn are essential metals and needed at low levels for normal biologic functions, whereas Cd, Cs, Hg, Ni, Pb, and Sn are nonessential metals that have no biologic function and pose toxicologic effects on living organisms. The resulting units were ng/mL. Metal concentrations below the limit of detection (LOD) were imputed with LOD/sqrt2 (Table S2; http://links.lww.com/EE/A190).

### Blood CRP and CAMs

CRP and CAMs were quantified using a customized Luminex assay from Invitrogen following the manufacturer’s recommended protocol, modified to include overnight incubation, with shaking, at 4°C. ICAM (Catalog no. EPX01A10201901) and VCAM (Catalog no. EPX01A10232901) were diluted 200-fold before running the assay, and CRP (Catalog no. EPX01A10288901) required a 2000-fold dilution. A Luminex-200 plate reader using xPonent software was used to acquire the raw data, which were compiled using Milliplex analyst (5.1.0.0). Samples were all run in duplicate, and the duplicate measures were averaged to arrive at a final concentration. All assays were performed at the Rogel Cancer Center’s Immune Monitoring Shared Resource Center at the University of Michigan (Table S1; http://links.lww.com/EE/A190). Laboratory methods were described in detail previously.^36,37^

An eight parameter standard curve, utilizing standard concentrations provided by the manufacturer, was used to convert optical density values into concentrations (pg/mL). The highest standard concentration was eliminated based on percent recovery being outside the acceptable range of 70% to 130% and standard curves were refit using the lower seven standards. Coefficients of variation (CVs) were then calculated, and samples with CVs above 30% were eliminated from all subsequent analyses. Intra-assay CVs ranged from 3.7% (VCAM) to 14.9% (CRP), and inter-assay CVs ranged from 7.9% (ICAM) to 12.0% (VCAM). All measured concentrations fell within the analytical limits of detection.

### Statistical analysis

Our study population initially consisted of 809 women (providing 1,098 samples) for whom we had biomarker data on at least one metal-CAMs pair. Various potential covariates were explored among this subset of women: maternal age, education level attained, marital status, employment, annual household income, smoking and exposure to secondhand smoke, alcohol use, parity, prepregnancy body mass index (BMI), and fetal sex. We utilized a forward stepwise procedure to add possible covariates into statistical models, which were retained in models if they resulted in a change in the main effect estimate by at least 10%. Resulting models adjusted for continuous maternal age and categorical forms of maternal education, exposure to environmental tobacco smoke, and prepregnancy BMI. After removing women with incomplete data on selected covariates (missing maternal education: 9; missing exposure to secondhand smoke: 65; missing prepregnancy BMI: 51; missing fetal sex: 115), our final sample consisted of 617 women who provided 857 blood samples (478 at visit 1 and 379 at visit 3).

Distributions of blood metal and CRP/CAM concentrations were assessed at each study visit. Intraclass correlation coefficients (ICCs), which describe between- and within-individual variability in biomarker concentrations across study visits, were also calculated. ICCs range between 0 (no reproducibility) and 1 (perfect reproducibility) and reflects a degree of reliability (<0.40: poor reliability, 0.40 < 0.75: moderate to good reliability, >0.75: excellent reliability).^38^ ICC estimates and their 95% confident intervals were calculated in R (version 4.0.4) using the package *ICC*. All blood biomarkers were log-normally distributed and thus were natural log-transformed for all subsequent analyses.

### Single-pollutant models

Linear-mixed effects models (LMEs) were used to regress CRP and CAMs on metals and included random intercepts for study participants to account for correlated repeated outcome measures. We then conducted additional analyses to explore possible effect modification by fetal sex and study visit on associations between metals and CRP/CAMs. Fetal sex and visit effects were estimated using interaction terms between metal exposure variables and fetal sex indicators, as well as study visit indicators. All results can be interpreted as the percent change in blood CRP/CAMs concentration with an interquartile range (IQR) increase in blood metal concentration. Using the Benjamini and Hochberg method,^39^ we calculated *q* values to address any potential false-positive results (*q* value > 0.2: a greater risk of being false-positives, *q* value < 0.2: a lower risk of being false-positives). Each inflammatory biomarker was treated as a family of tests (total of 11 tests with metals per inflammatory biomarker; 22 tests for sex- and visit-specific results). The significance level was set to alpha = 0.05.

## Results

Table 1 displays the demographic and health characteristics of the study participants. The mean age of the participants at the time of enrollment was 26.9 years (standard deviation of 5.5 years). About 80% of the study participants were either married or cohabitating (missing n = 2). A majority of the participants had earned tertiary education (80.9%), an annual household income of less than $50,000 (87.5%) (missing n = 65), and approximately 62% of the participants were employed at the time of enrollment (missing n = 7). About 50% of the study participants had a prepregnancy BMI of less than 25 kg/m^2^ (50.4%) and most of the participants did not smoke (98.8%) or drink (92.8%) (missing n = 2) during pregnancy. Fetal sex was evenly distributed among female (n = 294, 47.6%) and male infants (n = 323, 52.3%).

**Table 1.** Demographic and health characteristics of 617 women in the PROTECT birth cohort

Maternal age (yrs)	N (%)	Annual household income (dollar, $)	N (%)
18–24	224 (36.3)	<10k	176 (31.9)
25–29	193 (31.3)	10–<30k	179 (32.4)
30–34	129 (20.9)	30–<50k	128 (23.2)
35–41	71 (11.5)	≥50k	69 (12.5)
Missing	0	Missing	65
Maternal education	N (%)	Alcohol use	N (%)
GED or less	118 (19.1)	Never	293 (47.6)
Some college	219 (35.5)	Yes, before pregnancy	278 (45.2)
Bachelors or higher	280 (45.4)	Yes, currently	44 (7.2)
Missing	0	Missing	2
Marital status	N (%)	Number of children	N (%)
Single	120 (19.5)	0	265 (42.9)
Married	341 (55.4)	1	226 (36.6)
Cohabitating	154 (25.0)	2–5	126 (20.4)
Missing	2	Missing	0
Smoking status	N (%)	BMI	N (%)
Never	524 (84.9)	0, 25	311 (50.4)
Ever	86 (13.9)	25, 29.9	172 (27.9)
Current	7 (1.1)	29.9, 51	134 (21.7)
Missing	0	Missing	0
Currently employed	N (%)	Fetal sex	N (%)
No	229 (37.5)	Female	294 (47.6)
Yes	381 (62.5%)	Male	323 (52.4)
Missing	7	Missing	0
ETS	N (%)		
Never	561 (90.9)		
1 h	21 (3.4)		
>1 h	35 (5.7)		
Missing	0		

BMI indicates body mass index; ETS, environmental tobacco smoke; GED, general educational development.

Table S1 (http://links.lww.com/EE/A190) presents the maternal blood concentrations of CRP and CAMs. Both CRP and CAMs were measured above the LOD for all samples. CRP had the highest concentrations (median 3.86 mg/L and 3.76 mg/L for visits 1 and 3, respectively), and VCAM had the lowest concentrations (median 0.26 mg/L for both visits 1 and 3). Differing degrees of within- to between-participant variability were observed among CRP, ICAM, and VCAM (ICC values = 0.68, 0.88, 0.39, respectively), where ICAM showed the greatest temporal reliability and VCAM the lowest (Table S1; http://links.lww.com/EE/A190).

Table S2 (http://links.lww.com/EE/A190) shows the maternal blood metal concentrations including Cd, Co, Cs, Cu, Hg, Mn, Mo, Ni, Pb, Sn, and Zn. The majority of metals analyzed were above the LOD in at least 88% of samples. The only exception was Cd, which was measured above the LOD in 60% of samples. Of the essential metals, Zn (median visit 1: 4,648 ng/mL, visit 3: 4,823 ng/mL) concentrations were above the estimated reference range of blood zinc levels in pregnant women (Zn second trimester: 510–800 ng/mL, third trimester: 500–770 ng/mL).^40^ Also, we did not observe any toxic concentrations of nonessential metals in the study participants. In addition, Cu and Zn had a significant variance in their measurements between two study visits within participants as shown by their low ICC values of –0.02 and 0.10, respectively. Co, Mn, and Ni also had low temporal reliability shown by their low ICC values of 0.19, 0.16, and 0.26, respectively. Conversely, Mo and Sn levels had moderately high-temporal reliability (ICC values= 0.65, 0.68, respectively).

The associations between CRP and CAMs with maternal blood metal concentrations are shown in Table 2. An IQR increase in blood Ni was associated with higher blood CRP (%Δ = 7.04, 95% CI = 0.75, 13.73), and an IQR increase in blood Pb was associated with higher blood VCAM (%Δ = 4.57, 95% CI = 1.36, 7.89). ICAM had no significant associations with maternal blood metal concentrations in these models (Table 2).

**Table 2.** Results from linear-mixed effects models depicting the percent change in blood CRP/CAM concentrations with an interquartile range increase in blood metal concentration

	CRP				ICAM				VCAM			
	N	Est (95% CI)	*P*	*q*	N	Est (95% CI)	*P*	*q*	N	Est (95% CI)	*P*	*q*
Cd	731	–6.79 (–16.5, 4.04)	0.212	0.556	796	0.75 (–3.08, 4.74)	0.705	0.763	805	2.01 (–1.75, 5.91)	0.300	0.74
Co	731	–0.27 (–6.31, 6.16)	0.933	0.972	796	1.61 (–0.46, 3.71)	0.129	0.758	805	2.14 (–0.12, 4.45)	0.066	0.36
Cs	731	0.79 (–5.04, 6.96)	0.797	0.972	796	–0.61 (–2.60, 1.43)	0.557	0.763	805	–0.04 (–2.10, 2.07)	0.972	0.972
Cu	731	–0.66 (–1.87, 0.56)	0.288	0.556	796	0.12 (–0.27, 0.52)	0.536	0.763	805	–0.15 (–0.61, 0.30)	0.510	0.74
Hg	731	–0.15 (–8.31, 8.73)	0.972	0.972	796	–0.46 (–3.43, 2.59)	0.763	0.763	805	–1.17 (–3.94, 1.67)	0.416	0.74
Mn	731	–2.25 (–6.38, 2.06)	0.303	0.556	796	1.08 (–0.34, 2.51)	0.138	0.758	805	1.05 (–0.52, 2.64)	0.194	0.71
Mo	280	5.90 (–2.94, 15.6)	0.202	0.556	324	–1.13 (–3.93, 1.76)	0.441	0.763	329	–0.63 (–3.32, 2.13)	0.651	0.74
Ni	731	7.04 (0.75, 13.7)	0.029	0.32	796	0.69 (–1.30, 2.73)	0.501	0.763	805	–0.52 (–2.60, 1.60)	0.630	0.74
Pb	731	–4.18 (–13.0, 5.54)	0.387	0.609	796	0.85 (–2.79, 4.62)	0.653	0.763	805	4.57 (1.36, 7.89)	0.005	0.06
Sn	280	3.60 (–10.9, 20.4)	0.646	0.889	324	–2.73 (–7.53, 2.32)	0.287	0.763	329	–0.99 (–5.43, 3.66)	0.673	0.74
Zn	731	–1.45 (–3.25, 0.38)	0.122	0.556	796	0.12 (–0.49, 0.73)	0.709	0.763	805	–0.19 (–0.87, 0.49)	0.579	0.74

Models adjusted for continuous maternal age and categorical forms of maternal education, exposure to environmental tobacco smoke, and prepregnancy BMI. Models also included random intercepts for study participant.

Cd indicates cadmium; CI, confidence interval; Co, cobalt; CRP, c-reactive protein; Cs, cesium; Cu, copper; Est, effect estimate; Hg, mercury; ICAM, intercellular adhesion molecule; Mn, manganese; Mo, molybdenum; Ni, nickel; Pb, lead; Sn, tin; VCAM, vascular cell adhesion molecule; Zn, zinc.

**Figure 1.**
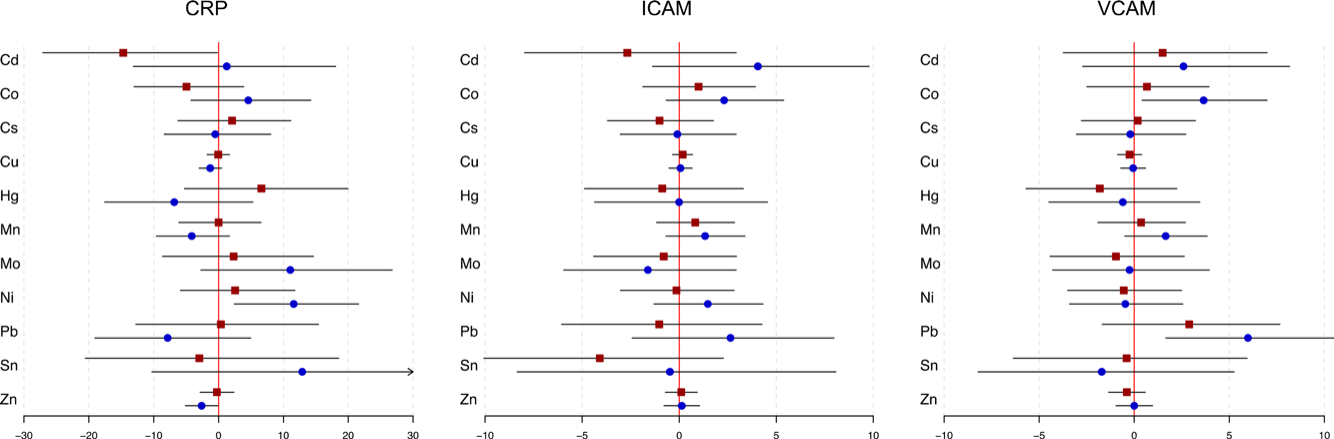
Effect estimates and 95% confidence intervals from linear-mixed effects models for the associations between blood metals and CRP/CAMs among 617 women in PROTECT, by fetal sex. Red squares denote estimates for female fetuses (n = 294) and blue circles denote estimates for male fetuses (n = 323).

When considering fetal sex in the models, we observed fetal sex-specific differences in associations between CRP and CAMs with maternal blood metal concentrations as shown in Figure 1 (corresponding effect estimates and confidence intervals are shown in Table S3; http://links.lww.com/EE/A190). Blood Cd was associated with a 14.7% decrease in CRP concentration (95% CI = –27.08, –0.19) among mothers carrying female fetuses. Conversely, blood Ni and Zn levels were associated (positively and negatively, respectively) with CRP concentration (Ni %Δ = 11.6, 95% CI = 2.41, 21.6; Zn %Δ = –2.61, 95% CI = –5.11, –0.05) only in mothers carrying male fetuses. Mo was also suggestively associated with increased CRP among male pregnancies. VCAM was positively associated with blood Co (%Δ = 3.65, 95% CI = 0.41, 6.98) and Pb (%Δ = 5.98, 95% CI = 1.66, 10.5) among mothers carrying male fetuses, but there were no sex-specific differences observed in ICAM.

**Figure 2.**
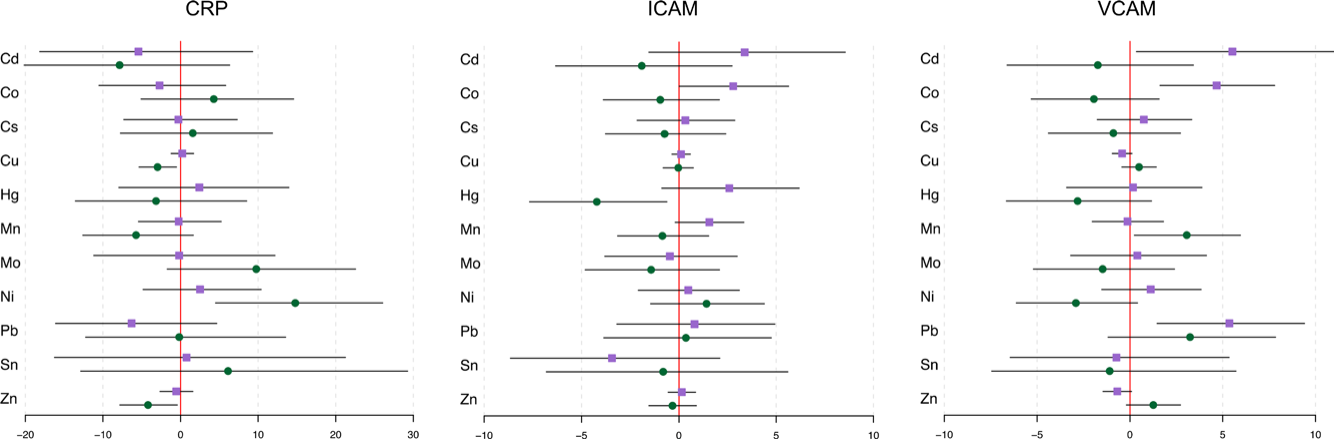
Effect estimates and 95% confidence intervals from linear-mixed effects models for the associations between blood metals and CRP/CAMs among 617 women in PROTECT, by study visit. Purple squares denote estimates for visit 1 (Cd, Co, Cs, Cu, Hg, Mn, Ni, Pb, Zn n = 478; Mo, Sn n = 187) and green circles denote estimates for visit 3 (Cd, Co, Cs, Cu, Hg, Mn, Ni, Pb, Zn n = 379; Mo, Sn n = 149).

Finally, we observed visit-specific differences in associations between CRP and CAMs with maternal blood metal concentrations (Figure 2). The time of the study visit played a significant role in the association between CRP and blood Cu and Ni concentration as indicated by the *P* value for the interaction term shown in Table S4; http://links.lww.com/EE/A190 (Cu p-int: 0.031; Ni p-int: 0.053). There were no significant associations between maternal blood concentrations with CRP at visit 1; however, blood Cu (%Δ = –2.97, 95% CI = –5.35, –0.53) and Zn (%Δ = –4.20, 95% CI = –7.83, –0.42) had significant negative associations, and Ni (%Δ = 14.78, 95% CI = 4.51, 26.05) had a significant positive association, with CRP at visit 3. Similarly, visit-specific differences were observed in both CAMs. Notably, the time of the study visit played a significant role in the association between ICAM and blood Hg concentration (p-int: 0.001), and in the relationships between VCAM and blood Cd (p-int: 0.042), Co (p-int: 0.005), and Zn concentrations (p-int: 0.021). An IQR increase in blood Co was associated with a 2.79% increase (95% CI = 0.01, 5.64) in ICAM concentration at visit 1, whereas blood Hg was associated with a 4.21% decrease (95% CI = –7.67, –0.62) in ICAM concentration at visit 3. VCAM had significant positive associations with blood Cd (%Δ = 5.52, 95% CI = 0.35, 11.0), Co (%Δ = 4.67, 95% CI = 1.62, 7.80), and Pb (%Δ = 5.36, 95% CI = 1.46, 9.41) at visit 1. Conversely, only blood Mn (%Δ = 3.06, 95% CI = 0.24, 5.96) had a significant association with maternal VCAM concentration at visit 3.

## Discussion

Multiple inflammatory events occur during pregnancy and especially during implantation, placentation, and preparation for delivery.^41^ CRP and CAMs are important regulators of inflammatory processes.^7,8^ Even though their exact roles in normal pregnancy remain unclear, studies have shown that elevated levels of these inflammatory regulators are associated with adverse birth outcomes.^18-27^ Notably, Lohsoonthorn et al^23^ found a relationship between high levels of CRP and increased risk of preterm birth in a cohort of 1,769 participants of the Omega study (Washington State). Similarly, Farzadnia et al observed severe preeclampsia in women with elevated serum levels of ICAM and VCAM in a cohort of 116 women in Iran (40 normal pregnant, 75 pregnant with preeclampsia). There is evidence, including human studies, demonstrating a link between heavy metal exposure and expression of CRP and CAMs,^42-44^ but associations specifically during pregnancy need to be further elucidated. Here, our current study further explored associations between metal exposures and maternal blood CRP and CAMs concentrations among pregnant women, as well as fetal sex-specific and gestational timing-specific differences in these associations.

Only limited human studies have demonstrated a link between prenatal metal exposure and an increase in maternal inflammatory markers.^45-49^ Notably, Farzan et al^45^ observed a relationship between prenatal arsenic exposure and an increase in cord blood of ICAM1 and VCAM1 in the New Hampshire Birth Cohort (n = 563). Similarly, in the present study, we observed significant positive associations between Ni and CRP and between Pb and VCAM (Table 2). Recently, Yuan et al^50^ measured blood metals and CRP concentrations among the Dongfeng-Tongji cohort adult population (n = 2,882, mean age of 65.58 years) and observed a positive association between CRP and blood Cu, but not with blood Ni. However, similar to our observations, blood Co, Mn, Mo, Pb, and Zn concentrations had no significant associations with maternal CRP concentrations.^50^ Importantly, the study suggested a metal-gene interaction by demonstrating blood Cu increasing the overall genetic predisposition to increased CRP level.^50^ However, age modulates gene expression.^51^ Therefore, it is unclear whether metal-gene interaction is the underlying cause of our observation due to an age-group difference between the cohorts. Furthermore, dramatic physiologic changes that occur during normal pregnancy can increase maternal susceptibility to prenatal exposures. For example, Pb tends to be accumulated in the bones and it is released throughout the body with calcium during pregnancy, resulting in increased maternal blood Pb concentration, as well as risk for developing pregnancy complications.^41^ Therefore, the conflicting results may be due to different maternal blood metal concentrations between the cohorts, leading to different effects on inflammatory biomarkers during pregnancy. It is also important to note that we calculated q values using the Benjamini and Hochberg^39^ to address the issues of false-positive associations. We observed that the Ni-CRP association has a higher *q* value (*q* = 0.32), suggesting greater risk of being false-positives. However, the Pb-VCAM association had a very low *q* value (*q* = 0.06), providing greater confidence in that association. More investigations are required to delineate possible mechanisms of the differential impact of metals on CRP and CAMs in the PROTECT cohort.

Fetal sex was used as an effect modifier in our study. The significant positive associations between Ni and CRP and between Pb and VCAM were mainly driven by mothers carrying male fetuses (Figure 2 and Table S3; http://links.lww.com/EE/A190). There are several studies that support fetal sex-specific immunological adaptation of pregnancy.^53-56^ Many studies have consistently demonstrated greater proinflammatory responses with a male fetus and greater anti-inflammatory responses with a female fetus,^53, 57-60^ resulting in a more protected environment with a female fetus than with a male fetus. Recently, Hunter et al^61^ observed that the male fetal-placental unit is more sensitive to maternal inflammation, including higher maternal CRP concentrations during gestation than female fetuses. Further, Al-Qaraghouli and Fang^52^ suggested that there are maternal-placental-fetal interactions that are fetal sex-specific. Therefore, more investigations are required to delineate the role of fetal sex on the impact of inflammatory cytokine levels and their relation to environmental toxicant-induced adverse birth outcomes.

Also, there are dynamic changes in the inflammatory profile during normal pregnancy.^15-17^ Therefore, the time of visit was also used as an effect modifier in our study, and we observed visit-specific associations between maternal blood concentration and CRP/CAMs. Notably, CRP had a positive association with Ni specifically at visit 3. Further, positive associations were observed between Cd, Co, and, Pb with VCAM, as well as between Co and ICAM at visit 1, but not at visit 3. Although the exact role of CRP and CAMs in pregnancy is not yet defined, there is evidence that suggests VCAM may play a role in the placentation process during early pregnancy.^62^ Also, Wirestam et al^63^ observed increased plasma CRP levels (median: 4.12 mg/L, *P* < 0.0001) in the third trimester (n = 100) during which necessary preparation for nascency occurs by triggering a proinflammatory response. These dynamic changes to the immune system during normal pregnancy may increase maternal susceptibility to prenatal metal exposures, resulting in visit-specific associations between blood metal concentrations and inflammatory biomarkers. However, there are differences in the methods used to measure these markers across studies; therefore, more investigations are required to further understand the role of CAMs, as well as CRP, in pregnancy, and large human studies measuring these markers at multiple time points in pregnancy will help inform future efforts.

There are important limitations in our study that need to be addressed. One limitation is that the blood samples in this study were only obtained from up to two study visits (median 18 and 26 weeks gestation). Inflammatory cytokines change dramatically during pregnancy.^56^ Therefore, using a blood sample only from two time points prevents a more detailed picture of the dynamic changes in CRP and CAMs over the course of pregnancy in our participants. It is also important to highlight that blood metal concentrations and inflammatory markers in our study were measured at the same time; therefore, we can not rule out the possibility of reverse causation. Further, there is risk of false-positive associations when conducting multiple statistical tests. Therefore, *q* values should be calculated to address the issues. Also, CRP/CAMs measurements alone cannot encompass the entirety of an inflammatory response, which might predict adverse birth outcomes. This is partly due to the interplay between inflammatory cytokines.^9-11^ Incorporating additional inflammatory cytokines, such as IL-1β and TNFα, and collecting measurements at additional time points, would be valuable in future studies. Finally, our study focused on an underrepresented community in the United States; therefore, the generalizability of these observations to other populations may be limited.

Despite these limitations, it is important to highlight the strengths of our study. There is limited research to date on the associations between metal exposure with CRP and CAMs in the context of pregnancy, and this study presents the association between maternal blood metal and CRP/CAMs levels. Even though we observed significant associations between CRP and VCAM with Ni and Pb, respectively, it is important to note that the majority of the metals, as well as all the inflammatory markers that we measured, were at low levels, as compared to their respective estimated reference values.^29,40,64^ There is increasing evidence of adverse pregnancy outcomes even at low levels of metal exposure.^65-72^ For example, Ashrap et al^30^ and Perkins et al^67^ reported that blood Pb levels even at low levels may be associated with preterm birth, which highlights the significance of our study. Furthermore, we observed fetal sex-specific, as well as visit-specific differences in associations between maternal blood metal concentrations and CRP/CAMs. Additionally, we conducted this analysis in an established and well-characterized birth cohort among an underrepresented population of pregnant women at risk for elevated environmental exposures as well as adverse birth outcomes.

## Conclusions

In this study, we present new information that may contribute to our understanding of relevant biologic pathways for adverse birth outcomes in pregnant women who are exposed to environmental contaminants. We observed significant associations between maternal blood metal levels and CRP/CAMs profiles in pregnant mothers in the PROTECT cohort. We also reported the impacts of fetal sex and timing of visits on metal associations with CRP/CAMs levels. Future work is warranted to explore metal associations with other inflammatory markers and their relation to adverse birth outcomes.

## Acknowledgments

We thank the nurses and research staff who participated in cohort recruitment and follow up, as well as the Federally Qualified Health Centers (FQHC) and clinics in Puerto Rico who facilitated participant recruitment, including Morovis Community Health Center (FQHC), Prymed: Ciales Community Health Center (FQHC), Camuy Health Services, Inc. (FQHC), and the Delta OBGyn (Prenatal Clinic).

## Conflicts of interest statement

The authors declare that they have no conflicts of interest with regard to the content of this report.

## Ethics statement

This study was approved by the research and ethics committees of the University of Puerto Rico, Northeastern University, and participating hospitals and clinics. The patients/participants provided their written informed consent to participate in this study.

## Supplementary Material


