# Supplementary material for: Maternal blood metal concentrations are associated with C-reactive protein and cell adhesion molecules among pregnant women in Puerto Rico
Source: Environ Epidemiol. 2022 Jul 5;6(4):e214. doi: 10.1097/EE9.0000000000000214 (PMC9374188; doi:10.1097/EE9.0000000000000214)
Supplement: Supplementary file 1 [file ee9-6-e214-s001.docx]

**Table S1**. Distributions of blood concentrations of CRP and CAMs (mg/L) among 617 women in the PROTECT birth cohort.

| **Biomarker** | **Visit** | **N** | **N>LOD** | **Min** | **p25** | **Med** | **p75** | **p90** | **Max** | **GM** | **IQR** | **ICC (95% CI)** |
| --- | --- | --- | --- | --- | --- | --- | --- | --- | --- | --- | --- | --- |
| CRP | 1 | 408 | 408 | 0.13 | 1.93 | 3.86 | 7.11 | 11.4 | 22.7 | 3.40 | 5.18 | 0.68 (0.60, 0.74) |
|  | 3 | 323 | 323 | 0.13 | 1.87 | 3.76 | 6.97 | 12.1 | 53.8 | 3.37 | 5.10 |  |
| ICAM | 1 | 445 | 445 | 0.09 | 0.50 | 0.61 | 0.77 | 1.10 | 3.87 | 0.64 | 0.27 | 0.88 (0.85, 0.90) |
|  | 3 | 351 | 351 | 0.22 | 0.50 | 0.61 | 0.81 | 1.24 | 9.98 | 0.67 | 0.31 |  |
| VCAM | 1 | 446 | 446 | 0.04 | 0.21 | 0.26 | 0.31 | 0.37 | 0.79 | 0.25 | 0.10 | 0.39 (0.27, 0.49) |
|  | 3 | 359 | 359 | 0.09 | 0.22 | 0.26 | 0.31 | 0.38 | 1.44 | 0.26 | 0.10 |  |
| All biomarkers were measured above the limit of detection (LOD) in 100% of samples. All concentrations are presented in mg/L. Abbreviations: CRP: C-reactive protein; ICAM: intercellular adhesion molecule; VCAM: vascular cell adhesion molecule; LOD: limit of detection; Min: minimum; Med: median; Max: maximum; GM: geometric mean; IQR: interquartile range; ICC: intraclass correlation coefficient; CI: confidence interval. | | | | | | | | | | | | |

**Table S2**. Distributions of blood metal concentrations among 617 women in the PROTECT birth cohort.

| **Metal** | **Visit** | **N** | **N>LOD** | **%>LOD** | **Min** | **p25** | **Med** | **p75** | **p90** | **Max** | **GM** | **IQR** | **ICC (95% CI)** |
| --- | --- | --- | --- | --- | --- | --- | --- | --- | --- | --- | --- | --- | --- |
| Cd | 1 | 478 | 285 | 59.6% | 0.07 | 0.07 | 0.11 | 0.17 | 0.23 | 1.68 | 0.12 | 0.10 | 0.52 (0.43, 0.60) |
|  | 3 | 379 | 238 | 62.8% | 0.07 | 0.07 | 0.12 | 0.17 | 0.23 | 1.34 | 0.12 | 0.10 |  |
| Co | 1 | 478 | 467 | 97.7% | 0.14 | 0.27 | 0.32 | 0.39 | 0.52 | 1.96 | 0.34 | 0.12 | 0.19 (0.05, 0.31) |
|  | 3 | 379 | 376 | 99.2% | 0.14 | 0.33 | 0.38 | 0.47 | 0.57 | 2.64 | 0.40 | 0.15 |  |
| Cs | 1 | 478 | 477 | 99.8% | 0.03 | 0.99 | 1.25 | 1.57 | 2.06 | 12.6 | 1.32 | 0.57 | 0.37 (0.25, 0.47) |
|  | 3 | 379 | 379 | 100% | 0.32 | 0.88 | 1.08 | 1.33 | 1.66 | 11.4 | 1.11 | 0.45 |  |
| Cu | 1 | 478 | 475 | 99.4% | 5.34 | 1331 | 1515 | 1700 | 1896 | 2847 | 1075 | 370 | -0.02 (-0.19, 0.12) |
|  | 3 | 379 | 379 | 100% | 7.48 | 1402 | 1615 | 1798 | 2020 | 3798 | 1407 | 396 |  |
| Hg | 1 | 478 | 477 | 99.8% | 0.141 | 0.83 | 1.21 | 1.74 | 2.56 | 9.54 | 1.21 | 0.91 | 0.58 (0.49, 0.65) |
|  | 3 | 379 | 379 | 100% | 0.21 | 0.83 | 1.19 | 1.63 | 2.53 | 10.6 | 1.18 | 0.80 |  |
| Mn | 1 | 478 | 476 | 99.6% | 0.23 | 7.67 | 9.84 | 12.6 | 14.8 | 90.7 | 8.65 | 4.97 | 0.16 (0.02, 0.29) |
|  | 3 | 379 | 379 | 100% | 0.55 | 9.82 | 12.2 | 15.2 | 18.1 | 34.9 | 11.4 | 5.40 |  |
| Mo | 1 | 187 | 187 | 100% | 0.43 | 39.9 | 55.8 | 80.3 | 115 | 229 | 46.9 | 40.4 | 0.65 (0.51, 0.74) |
|  | 3 | 149 | 149 | 100% | 0.39 | 26.7 | 43.3 | 69.0 | 101 | 274 | 35.9 | 42.4 |  |
| Ni | 1 | 478 | 445 | 93.1% | 0.35 | 0.80 | 1.02 | 1.39 | 2.28 | 22.8 | 1.13 | 0.59 | 0.26 (0.12, 0.37) |
|  | 3 | 379 | 350 | 92.3% | 0.35 | 0.79 | 1.02 | 1.35 | 1.83 | 7.8 | 1.03 | 0.55 |  |
| Pb | 1 | 478 | 456 | 95.4% | 0.01 | 0.23 | 0.32 | 0.42 | 0.52 | 2.18 | 0.31 | 0.18 | 0.74 (0.68, 0.78) |
|  | 3 | 379 | 373 | 98.4% | 0.11 | 0.24 | 0.32 | 0.43 | 0.56 | 1.51 | 0.33 | 0.19 |  |
| Sn | 1 | 187 | 166 | 88.8% | 0.07 | 0.51 | 1.29 | 2.80 | 6.28 | 25.0 | 1.24 | 2.29 | 0.68 (0.55, 0.76) |
|  | 3 | 149 | 128 | 85.9% | 0.07 | 0.51 | 1.17 | 2.31 | 4.78 | 133 | 1.07 | 1.80 |  |
| Zn | 1 | 478 | 477 | 99.8% | 13.6 | 4061 | 4648 | 5214 | 5730 | 7840 | 3761 | 1153 | 0.10 (-0.05, 0.23) |
|  | 3 | 379 | 379 | 100% | 131 | 4299 | 4823 | 5301 | 5725 | 8043 | 4419 | 1002 |  |
| All concentrations are presented in ng/mL. Abbreviations: Cd: cadmium; Co: cobalt; Cs: cesium; Cu: copper; Hg: mercury; Mn: manganese; Mo: molybdenum; Ni: nickel; Pb: lead; Sn: tin; Zn: zinc; LOD: limit of detection; Min: minimum; Med: median; Max: maximum; GM: geometric mean; IQR: interquartile range; ICC: intraclass correlation coefficient; CI: confidence interval. | | | | | | | | | | | | | |

**Table S3**. Effect estimates and 95% confidence intervals for associations between blood metal and CRP/CAM concentrations, by fetal sex, among 617 women in PROTECT. Estimates represent the percent change in CRP/CAM with an interquartile range increase in blood metal.

| CRP | | | | | |
| --- | --- | --- | --- | --- | --- |
|  | Females | q-value | P-Int | Males | q-value |
| Cd | **-14.7 (-27.1, -0.19)** | 0.358 | 0.127 | 1.26 (-13.1, 18.0) | 0.996 |
| Co | -4.95 (-13.0, 3.86) | 0.588 | 0.136 | 4.58 (-4.24, 14.2) | 0.588 |
| Cs | 2.07 (-6.25, 11.1) | 0.996 | 0.671 | -0.51 (-8.39, 8.05) | 0.996 |
| Cu | -0.05 (-1.75, 1.68) | 0.996 | 0.320 | -1.29 (-3.00, 0.45) | 0.588 |
| Hg | 6.60 (-5.27, 20.0) | 0.588 | 0.121 | -6.83 (-17.6, 5.29) | 0.588 |
| Mn | 0.02 (-6.12, 6.55) | 0.996 | 0.338 | -4.13 (-9.61, 1.68) | 0.588 |
| Mo | 2.33 (-8.64, 14.6) | 0.996 | 0.355 | 11.1 (-2.72, 26.8) | 0.588 |
| Ni | 2.55 (-5.90, 11.8) | 0.959 | 0.176 | **11.6 (2.41, 21.6)** | 0.289 |
| Pb | 0.36 (-12.7, 15.4) | 0.996 | 0.374 | -7.84 (-19.1, 4.94) | 0.588 |
| Sn | -2.96 (-20.5, 18.5) | 0.996 | 0.335 | 12.9 (-10.3, 42.1) | 0.588 |
| Zn | -0.26 (-2.83, 2.38) | 0.996 | 0.206 | **-2.61 (-5.11, -0.05)** | 0.358 |
| ICAM | | | | | |
|  | Females | q-value | P-Int | Males | q-value |
| Cd | -2.67 (-7.97, 2.93) | 0.963 | 0.092 | 4.05 (-1.37, 9.78) | 0.963 |
| Co | 0.99 (-1.87, 3.92) | 0.963 | 0.536 | 2.31 (-0.67, 5.38) | 0.963 |
| Cs | -1.01 (-3.69, 1.76) | 0.963 | 0.661 | -0.10 (-3.04, 2.93) | 0.992 |
| Cu | 0.17 (-0.35, 0.69) | 0.963 | 0.790 | 0.06 (-0.53, 0.66) | 0.998 |
| Hg | -0.87 (-4.88, 3.30) | 0.992 | 0.779 | -0.01 (-4.36, 4.55) | 0.998 |
| Mn | 0.82 (-1.16, 2.85) | 0.963 | 0.727 | 1.33 (-0.68, 3.38) | 0.963 |
| Mo | -0.80 (-4.41, 2.95) | 0.992 | 0.783 | -1.61 (-5.95, 2.93) | 0.963 |
| Ni | -0.15 (-3.03, 2.82) | 0.992 | 0.435 | 1.47 (-1.29, 4.31) | 0.963 |
| Pb | -1.03 (-6.06, 4.26) | 0.992 | 0.321 | 2.64 (-2.42, 7.97) | 0.963 |
| Sn | -4.09 (-10.1, 2.27) | 0.963 | 0.490 | -0.48 (-8.35, 8.07) | 0.992 |
| Zn | 0.10 (-0.71, 0.92) | 0.992 | 0.961 | 0.13 (-0.78, 1.05) | 0.992 |
| VCAM | | | | | |
|  | Females | q-value | P-Int | Males | q-value |
| Cd | 1.49 (-3.73, 6.99) | 0.952 | 0.778 | 2.59 (-2.71, 8.17) | 0.952 |
| Co | 0.67 (-2.49, 3.93) | 0.952 | 0.206 | **3.65 (0.41, 6.98)** | 0.305 |
| Cs | 0.18 (-2.78, 3.22) | 0.952 | 0.856 | -0.21 (-3.04, 2.71) | 0.952 |
| Cu | -0.25 (-0.88, 0.39) | 0.952 | 0.686 | -0.06 (-0.71, 0.60) | 0.952 |
| Hg | -1.81 (-5.70, 2.24) | 0.952 | 0.673 | -0.60 (-4.49, 3.45) | 0.952 |
| Mn | 0.36 (-1.92, 2.69) | 0.952 | 0.424 | 1.65 (-0.50, 3.84) | 0.952 |
| Mo | -0.96 (-4.43, 2.63) | 0.952 | 0.796 | -0.25 (-4.29, 3.95) | 0.952 |
| Ni | -0.55 (-3.50, 2.49) | 0.952 | 0.971 | -0.47 (-3.40, 2.55) | 0.952 |
| Pb | 2.89 (-1.68, 7.66) | 0.952 | 0.338 | **5.98 (1.66, 10.5)** | **0.147** |
| Sn | -0.40 (-6.36, 5.93) | 0.952 | 0.779 | -1.71 (-8.21, 5.25) | 0.952 |
| Zn | -0.39 (-1.35, 0.58) | 0.952 | 0.572 | 0.00 (-0.96, 0.97) | 0.999 |
| Models adjust for maternal age, education, exposure to secondhand tobacco smoke, and pre-pregnancy BMI. | | | | | |

**Table S4**. Effect estimates and 95% confidence intervals for associations between blood metal and CRP/CAM concentrations, by study visit, among 617 women in PROTECT. Estimates represent the percent change in CRP/CAM with an interquartile range increase in blood metal.

| CRP | | | | | |
| --- | --- | --- | --- | --- | --- |
|  | Visit 1 | q-value | P-Int | Visit 3 | q-value |
| Cd | -5.42 (-18.2, 9.29) | 0.957 | 0.782 | -7.86 (-20.2, 6.32) | 0.829 |
| Co | -2.70 (-10.5, 5.81) | 0.957 | 0.261 | 4.28 (-5.08, 14.6) | 0.957 |
| Cs | -0.28 (-7.32, 7.30) | 0.979 | 0.753 | 1.57 (-7.76, 11.8) | 0.979 |
| Cu | 0.22 (-1.22, 1.69) | 0.979 | **0.031** | **-2.97 (-5.35, -0.53)** | 0.201 |
| Hg | 2.42 (-7.95, 14.0) | 0.97 | 0.433 | -3.16 (-13.6, 8.51) | 0.979 |
| Mn | -0.24 (-5.43, 5.24) | 0.979 | 0.217 | -5.74 (-12.6, 1.65) | 0.558 |
| Mo | -0.19 (-11.2, 12.2) | 0.979 | 0.202 | 9.75 (-1.72, 22.6) | 0.558 |
| Ni | 2.50 (-4.83, 10.4) | 0.957 | 0.053 | **14.8 (4.51, 26.0)** | **0.097** |
| Pb | -6.31 (-16.1, 4.66) | 0.829 | 0.370 | -0.17 (-12.2, 13.5) | 0.979 |
| Sn | 0.77 (-16.3, 21.2) | 0.979 | 0.669 | 6.12 (-12.9, 29.3) | 0.957 |
| Zn | -0.55 (-2.64, 1.58) | 0.957 | 0.098 | **-4.20 (-7.83, -0.42)** | 0.228 |
| ICAM | | | | | |
|  | Visit 1 | q-value | P-Int | Visit 3 | q-value |
| Cd | 3.38 (-1.53, 8.55) | 0.801 | 0.062 | -1.90 (-6.33, 2.73) | 0.74 |
| Co | **2.79 (0.01, 5.64)** | 0.552 | 0.055 | -0.95 (-3.88, 2.08) | 0.553 |
| Cs | 0.33 (-2.14, 2.87) | 0.883 | 0.557 | -0.73 (-3.77, 2.41) | 0.809 |
| Cu | 0.11 (-0.36, 0.59) | 0.883 | 0.755 | -0.03 (-0.80, 0.74) | 0.556 |
| Hg | 2.59 (-0.87, 6.18) | 0.801 | **0.001** | **-4.21 (-7.67, -0.62)** | 0.363 |
| Mn | 1.56 (-0.19, 3.34) | 0.607 | 0.090 | -0.84 (-3.15, 1.53) | 0.205 |
| Mo | -0.46 (-3.80, 3.00) | 0.883 | 0.613 | -1.42 (-4.81, 2.09) | 0.714 |
| Ni | 0.48 (-2.08, 3.11) | 0.883 | 0.619 | 1.42 (-1.45, 4.39) | 0.282 |
| Pb | 0.80 (-3.18, 4.93) | 0.883 | 0.823 | 0.36 (-3.85, 4.75) | 0.363 |
| Sn | -3.43 (-8.65, 2.10) | 0.818 | 0.373 | -0.80 (-6.81, 5.60) | 0.912 |
| Zn | 0.15 (-0.55, 0.86) | 0.883 | 0.503 | -0.33 (-1.53, 0.90) | 0.282 |
| VCAM | | | | | |
|  | Visit 1 | q-value | P-Int | Visit 3 | q-value |
| Cd | **5.52 (0.35, 11.0)** | 0.205 | **0.042** | -1.73 (-6.62, 3.42) | 0.74 |
| Co | **4.67 (1.62, 7.80)** | **0.06** | **0.005** | -1.94 (-5.32, 1.57) | 0.553 |
| Cs | 0.75 (-1.76, 3.33) | 0.773 | 0.449 | -0.89 (-4.39, 2.73) | 0.809 |
| Cu | -0.42 (-0.95, 0.11) | 0.34 | 0.099 | 0.49 (-0.44, 1.42) | 0.556 |
| Hg | 0.17 (-3.41, 3.88) | 0.928 | 0.248 | -2.82 (-6.65, 1.17) | 0.363 |
| Mn | -0.13 (-2.03, 1.81) | 0.928 | 0.064 | **3.06 (0.24, 5.96)** | 0.205 |
| Mo | 0.40 (-3.19, 4.13) | 0.912 | 0.469 | -1.47 (-5.20, 2.41) | 0.714 |
| Ni | 1.12 (-1.52, 3.83) | 0.696 | 0.059 | -2.91 (-6.12, 0.41) | 0.282 |
| Pb | **5.36 (1.46, 9.41)** | **0.078** | 0.440 | 3.24 (-1.18, 7.85) | 0.363 |
| Sn | -0.73 (-6.45, 5.34) | 0.912 | 0.934 | -1.09 (-7.45, 5.71) | 0.912 |
| Zn | -0.68 (-1.45, 0.09) | 0.282 | **0.021** | 1.26 (-0.19, 2.73) | 0.282 |
| Models adjust for maternal age, education, exposure to secondhand tobacco smoke, and pre-pregnancy BMI. | | | | | |
